# Adaptively triggered comparisons enhance perceptual category learning: evidence from face learning

**DOI:** 10.1038/s41598-024-70163-6

**Published:** 2024-08-27

**Authors:** Victoria L. Jacoby, Christine M. Massey, Philip J. Kellman

**Affiliations:** 1 Department of Psychology, University of California Los Angeles, Los Angeles, CA USA; 2 Department of Surgery, David Geffen School of Medicine, University of California Los Angeles, Los Angeles, CA USA

**Keywords:** Perceptual learning, Adaptive learning, Categorization, Comparison, Face perception, Psychology, Human behaviour

## Abstract

Categorical learning is important and often challenging in both specialized domains, such as medical image interpretation, and commonplace ones, such as face recognition. Research has shown that comparing items from different categories can enhance the learning of perceptual classifications, particularly when those categories appear highly similar. Here, we developed and tested novel *adaptively triggered comparisons* (ATCs), in which errors produced during interactive learning dynamically prompted the presentation of active comparison trials. In a facial identity paradigm, undergraduate participants learned to recognize and name varying views of 22 unknown people. In Experiment 1, single-item classification trials were compared to a condition in which ATC trials were generated whenever a participant repeatedly confused two faces. Comparison trials required discrimination between simultaneously presented exemplars from the confused categories. In Experiment 2, an ATC condition was compared to a non-adaptive comparison condition. Participants learned to accuracy and speed criteria, and completed immediate and delayed posttests. ATCs substantially enhanced learning efficiency in both experiments. These studies, using a novel adaptive procedure guided by each learner’s performance, show that adaptively triggered comparisons improve category learning.

## Introduction

Classifying objects based on perceptual information is a requirement of many real-world learning tasks, such as identifying faces across varying conditions, identifying the artist who created a painting, or distinguishing cancerous skin lesions from benign ones. These abilities improve profoundly with experience, largely driven by processes of *perceptual learning* (PL)—experience-driven improvements in the pickup of information (for reviews, see^1–3^). While experts in a domain may *know* more than novices, much of their expertise comes from changed ways of *seeing*^4–6^. We emphasize visual PL here, but PL is also highly important in tasks or learning domains where information comes in through other senses, (for reviews, see^1,7^) or perceived properties that can be detected through more than one sense (e.g.,^8^). These changes include attunement of perceptual mechanisms to selectively pick up relevant information, discovery of relevant patterns and relations, and fluency changes, such as more rapid and automatic information extraction^3,9^. Contrary to some views that *perceptual learning* should refer only to sensory plasticity in the earliest cortical areas of each sense (e.g.,^10^), PL occurs at multiple levels and comprises a crucial component of expertise in many learning domains (for discussion of the scope and nature of PL, see^1,3,8,11^). When learning multiple perceptual classifications, PL attunes perceptual mechanisms to preferentially encode information that distinguishes different categories, as well as diagnostic features common to members of the same category^1^. These changes enable one of the most impactful outcomes of category learning: accurate and effortless categorization of novel instances.

In ordinary experience, the ability to learn categories and transfer learning to new instances appears to occur from exposure to exemplars that are encountered successively, often widely dispersed in time. This suggests that some attributes of experienced exemplars are encoded sufficiently for learners to come to utilize distinguishing features between and relevant commonalities among exemplars experienced at different times. However, there are strong reasons to suggest that discovery and extraction of these features can be accelerated through the direct comparison of items.

### Comparisons

Considerable research has shown that explicit comparisons benefit learning in general, (e.g.,^12,13^), and the acquisition and transfer of categories in particular (e.g.,^14,15^). Simultaneous comparisons—presentation of exemplars from the same or different categories—may allow PL processes to identify informational variables more readily by reducing dependence on long term memory. If PL is, at least in part, a filtering process wherein relevant information must be discovered or upweighted and irrelevant information downweighted^16^, simultaneous comparisons should allow easier discovery of commonalities and differences relevant to categorization. This advantage may be especially important in the concurrent learning of multiple categories in complex learning domains. Empirical results have provided support for this idea by demonstrating a learning advantage for categories that were presented concurrently alongside other items in learning relative to being presented in isolation^17–20^.

Prior research has shown that comparing items from different categories can enhance learning and transfer^17–19,21–23^, though the strength of these effects varies across different domains and categories. In particular, between-category comparisons are maximally beneficial when categories are highly similar and distinguishing characteristics are difficult to identify; conversely, when these categories are dissimilar, there may be little or no benefit^24,25^.

Given that PL leads to changes in the information extracted, the similarity of objects relative to each other can change throughout the course of learning^26–28^. This suggests that the value of comparing exemplars from two categories may vary as learning proceeds. Further, learners progress along different paths. Sensitivity to relevant features and category confusions may differ for each learner. Presenting diverse learners with comparisons of the same content or at the same points in learning may fail to tap the full potential of learning with comparisons. In the present work, we investigated this issue by testing an adaptive learning approach to comparisons that utilizes individual learning data to determine what comparisons get presented and when.

### Adaptive learning

Adaptive learning methods use individual learner responses to tailor events in learning in ways that benefit that specific learner. Much research in adaptive learning has focused on foreign language vocabulary or other factual domains. Specific individual items would recur during learning depending on a learner’s accuracy for that item on earlier presentations. In recent years, adaptive learning methods have been applied to perceptual learning in diverse domains, including natural categories^29^, mathematics and STEM learning^6,30^, and medical learning (for a review, see^31^). Note that our use of “adaptive learning” in this context refers to procedures that use learner performance data to create and guide the flow of events with the aim of *optimizing* learning. Adaptive learning methods as intended here usually have a different aim from adaptive methods for measurement in psychophysics, such as adaptive staircases^32,33^.

The latter use observer performance to *assess* learning (i.e., they vary stimulus values in order to estimate the value of a parameter in a psychometric function, such as threshold or slope). In fact, for adaptive methods used in parameter estimation, changes in performance due to learning pose special challenges because standard methods typically assume stationarity – that the parameter being estimated is not changing while being measured^34^. Whereas the goals for optimizing learning and assessing it are often distinct, it is possible that some existing or potential efforts might usefully combine these goals (e.g.,^35^).

Research efforts have consistently shown that PL can be enhanced using adaptive learning methods. Most of these efforts have utilized the Adaptive Response Time-Based Sequencing (ARTS) system^29,36,37^, which can be applied both to factual/declarative learning and to PL. Research on the spacing effect suggests that recurring practice with a given learning item should ideally occur when it is maximally difficult to recall, but has not yet been forgotten^29,38–40^. The ARTS system uses an algorithm based on accuracy, response times, and trials since last presentation to concurrently guide spacing and recurrence for all members of a set of learning items. Evidence indicates that these methods lead to better learning when compared to non-adaptive sequencing methods (e.g.,^37^).

When applied to PL, ARTS uses response times and accuracy to determine spacing and recurrence of each learning *category*. In contrast to most factual learning situations, where the same learning item reappears, recurrence of a category in PL involves presentation of novel instances.

In the present work, we investigated a new type of adaptive learning intervention, which we call *adaptively triggered comparisons (ATCs)*. ATCs are comparison trials whose timing and content are prompted by errors in which a learner confuses two categories. ATCs consider not only whether an item was classified correctly, but also the specific answer that was given for each incorrectly classified trial. New learning events (comparison trials) are then triggered by identifying patterns in the incorrect answers given for each category.

We chose the domain of face identification as an ecologically natural, often challenging domain, and one in which the relevance of perceptual learning is intuitive, demonstrated by considerable prior research^41–43^, and noted as an important topic in high-level perceptual learning in recent reviews of the field (e.g.,^44^). Although our primary focus is on investigating adaptive use of comparisons to enhance learning, which may have value across many learning domains, the study of this approach in face perception also has the potential to lead to practical applications in face learning for normal and prosopagnosic observers^41,45,46^. To study whether adaptive use of comparisons in face perception may provide learning advantages beyond those already given by an effective adaptive system, we applied ATCs in the context of an adaptive learning paradigm for face-identification as follows. If, during single-item classification trials, a learner was presented with a picture of David but responded “Alexander,” that information could be used to generate a simultaneous, between-category comparison trial in which pictures of David and Alexander appeared side by side. Intuitively, the value of ATCs might be twofold: tracking learners’ errors identifies specific confusions during learning, while simultaneous comparisons may help resolve them by facilitating the discovery of critical invariants that may be harder to detect when items appear in isolation.

Human faces share considerable overlap in perceptual features, often containing the same major elements configured in relatively consistent ways. As a result, learning to recognize unfamiliar faces is dependent upon the use of distinguishing information that is often subtle, complex, and difficult to verbalize^43^. These characteristics and considerable research (e.g.,^25,41–44^) implicate perceptual learning as a primary driver of improvement in face discrimination. Some prior research specifically indicates that perceptual learning of unfamiliar faces can also be improved through the use of simultaneous comparisons^25^. Specifically, presenting faces side by side may allow the observer to quickly adapt to the many common features shared across the simultaneously presented faces and subsequently emphasize the remaining distinguishing features of each face^47^. Consistent with influential models of perceptual learning (e.g.,^16^) reweighting of perceptual information in favor of distinctive features has been theorized to consequently modify representations of each face, enabling more accurate recognition in future encounters^25^.

Across two experiments, we tested whether learning could be improved through the inclusion of ATCs. The task involved PL rather than simple image memorization in that a person’s name was used as a category but different exemplars of the person’s face appeared upon presentations of that category. In both experiments, participants were trained to classify 22 different face categories. Experiment 1 compared performance using only single-item classification trials scheduled through ARTS (*No Comparisons Condition*) with a condition mixing single-item classification trials and ATC trials (*ATC Condition*). Experiment 2 asked whether the inclusion of adaptive comparison trials enhanced learning to a greater degree than learning with an equal number of non-adaptive simultaneous comparison trials (*Non-Adaptive Comparisons (NAC) Condition)*.

## Experiment 1

### Method

#### Participants

82 undergraduate psychology students were recruited from the University of California, Los Angeles to participate. Two participants were dropped for failure to follow experimenter instructions and an additional four were dropped for technical issues during the experiment. The remaining participants were equally distributed between the ATC condition (*n* = 38) and the No Comparisons condition (*n* = 38). Participants provided informed consent and received partial course credit for their participation. This study was approved by the UCLA Institutional Review Board. All methods were carried out in accordance with their guidelines and regulations.

#### Stimuli

**Figure 1:**
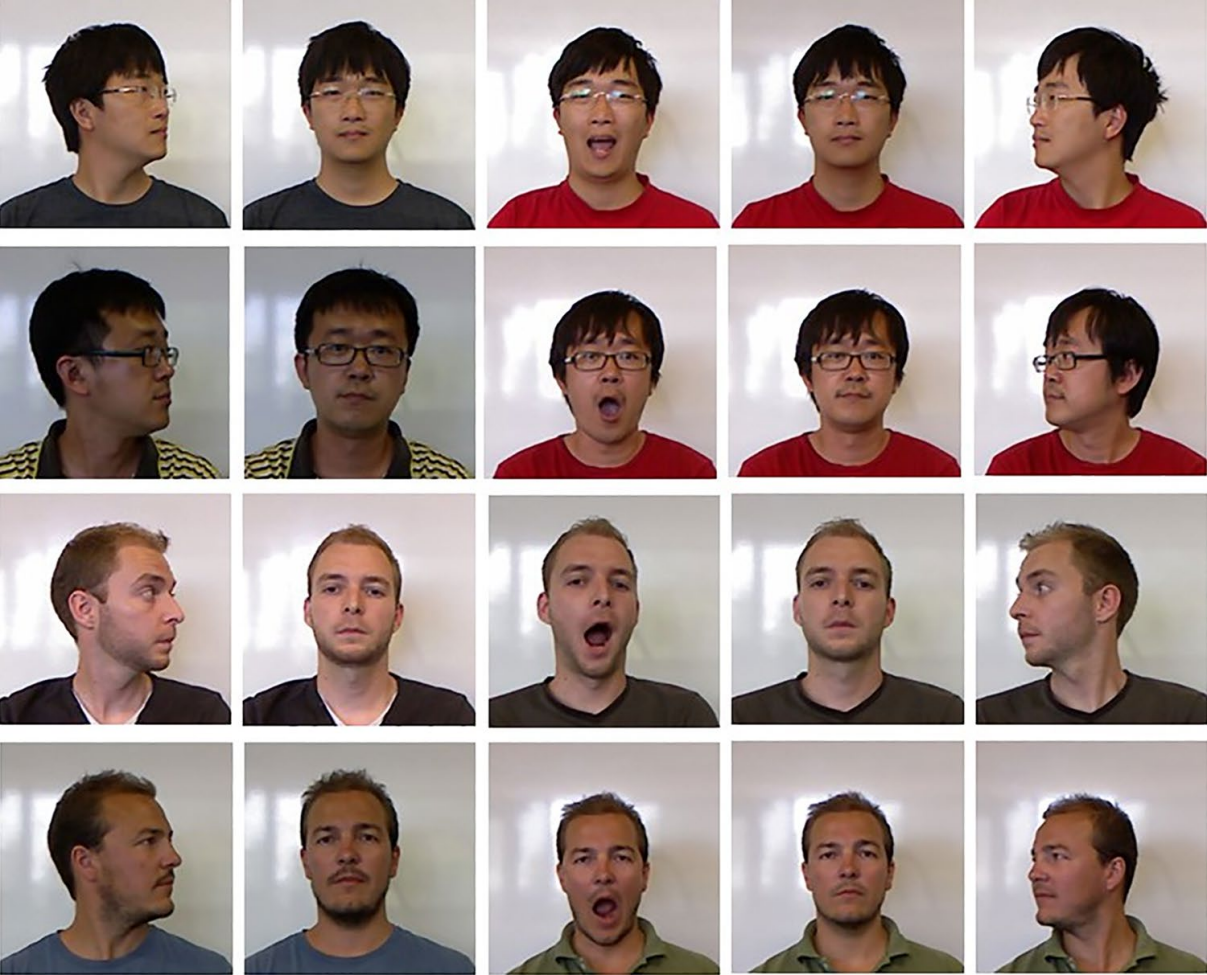
Example face categories. The five face images used for four of the 22 face categories are shown above. Each row contains one face category. All face images used in this study are publicly available through the EURECOM Kinect Face Dataset: https://rgb-d.eurecom.fr^48^.

We used 22 human male faces with five distinct pictures of each person for a total of 110 unique images taken from a larger database^48^. Examples of these categories can be seen in Fig. 1. Four images from each of the 22 categories were used in the learning phase. The fifth image in each category was set aside for use as a novel stimulus in the posttests. Non–face details such as hairstyle or visible clothing varied across images within the same category. Background and final image size (256 × 256 pixels) were the same for all images.

Each face category was identified with a name. The names were chosen to be unremarkable, and were taken from the Social Security list of most common names given in the United States in 2000–2009. Although our task contained a declarative learning component (the names), the use of different exemplars for each facial identity category, and the withholding of a novel exemplar of each for assessments, placed important demands on perceptual learning of facial characteristics to be designated by a name rather than attachment of a name to a single image. Evidence suggests both that recognition of different face images from a single individual can be challenging^49^, and that experience of variation is a highly useful means of coming to perceive facial identity^50,51^.

#### Design and procedure

Participants were randomly assigned to either the ATC condition or No Comparisons condition. All participants completed a learning phase followed by an immediate posttest. Delayed posttests were completed one week later.

**Figure 2:**
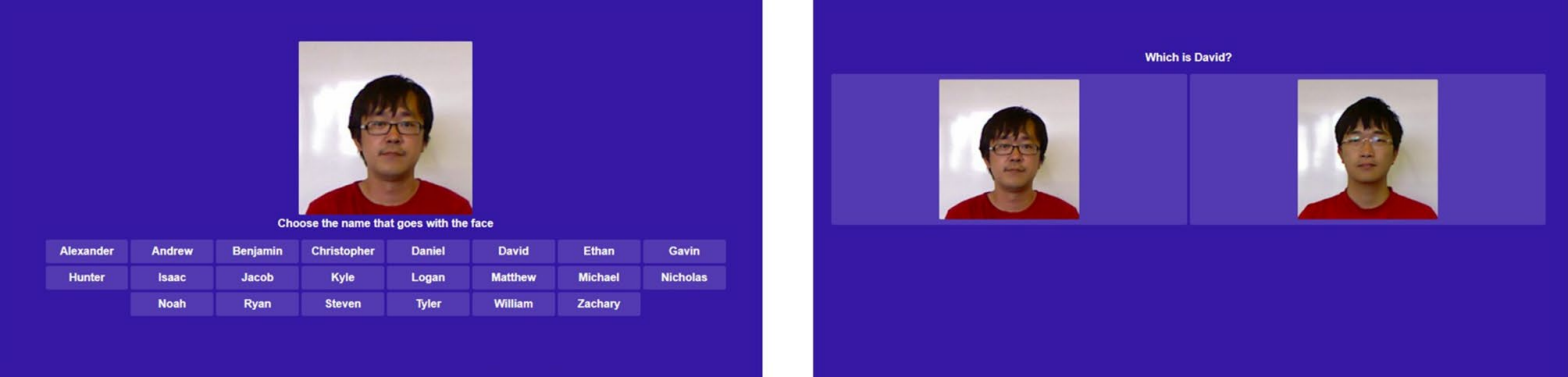
Example learning trials. The left panel depicts an example single-item classification learning trial (“Choose the name that goes with the face”). The right panel depicts an example comparison trial (“Which is [Category Name]?”). All face images used in this study are publicly available through the EURECOM Kinect Face Dataset: https://rgb-d.eurecom.fr^48^.

Figure 2 shows an example of the types of learning trials a participant could encounter. On most learning trials in either condition, a single image was presented with all 22 possible names shown alphabetically below. Participants were instructed to select the name that matched the presented picture. Participants had 20 s to answer and were given immediate feedback. Feedback indicated whether their given answer was correct or incorrect and displayed the phrase “This is [Category Name]” at the bottom of the screen.

In the ATC condition, participants’ answers on single-item learning trials were monitored for patterns that indicated systematic confusions. A confusion was defined as two incorrect answers that involved the same two categories. For example, for categories Alexander and Benjamin, a confusion would be registered if (a) an Alexander exemplar was incorrectly labeled “Benjamin” twice; (b) a Benjamin exemplar was incorrectly labeled “Alexander” twice; or c) an Alexander exemplar was incorrectly labeled “Benjamin” once and a Benjamin exemplar was incorrectly labeled “Alexander'' once. When a confusion was detected, it triggered the creation of a comparison trial in the ATC condition.

The choice of the number of errors needed to trigger a comparison trial was based on two primary factors. The first was the number of learning categories. With 22 facial identities to learn, we reasoned that this set was large enough that two confusions of the same identities would be relatively unlikely to occur by chance, thereby avoiding the triggering of confusions based on random guessing behavior. Second, we aimed to establish a criterion for triggering comparison trials that would be able to target confusions at an early stage and be met often enough to provide sufficient opportunity to engage with this intervention.

On a comparison trial, one randomly chosen exemplar from each of the two confused categories was presented with the prompt “Which is [Category Name]?” Participants were required to select the image that they believed matched the name provided. Feedback was given immediately with the appropriate category label displayed beneath each picture.

Comparison trials occurred at the next scheduled presentation of whichever of the two categories reappeared soonest in learning following the confusion. For example, if, following an Alexander-Benjamin confusion, “Benjamin” was scheduled to reappear before “Alexander”, then the next presentation of Benjamin would be in the context of an Alexander-Benjamin comparison trial rather than in a standard single-item classification trial. Given the way categories were sequenced throughout learning, this resulted in ATC trials occurring, on average, after a delay of 3 trials from the second misclassification. Following the completion of a comparison trial, participants then resumed single-classification trials. Another comparison trial between the two categories would be triggered only if the categories were again confused on a subsequent single-classification trial.

In both conditions, categories were adaptively scheduled and interleaved through the Adaptive Response Time-Based Sequencing (ARTS) system. During learning in ARTS, each category is assigned a priority score indicating the relative benefit of that category appearing on the next learning trial. Priority scores for a category are a function of learner accuracy, response times, trials elapsed since last presentation, and progress toward meeting mastery criteria (see^29,37,52^ for computational details). The sequencing algorithm presents the highest priority category on each trial. An enforced delay is also implemented: a category could not recur while feedback from a recent instance could still reside in working memory. In the present study, we used an enforced delay of 3 trials with a ± 1 jitter, such that the delay was sometimes 2 or 4 (25% each). As an individual’s learning strength for a given category increases (indicated by greater accuracy and lower RTs), the ARTS algorithm automatically generates lower priority, and longer recurrence intervals, as an inverse function of the logarithm of reaction time.

Participants continued learning trials until all categories reached mastery. Mastery criteria for each category required four consecutive correct classifications, each given in under five seconds. We used mastery criteria due to their importance in real-world learning settings and to ensure that participants in both conditions learned the full set of categories to an equivalent standard. Exemplars from all categories could appear in learning until the last category was mastered. Immediately following the learning phase, participants completed a 44-item posttest including one previously seen and one novel exemplar per category, randomized and presented sequentially. Posttest trials were the same as single-classification learning trials; however, no feedback was given. A delayed posttest, administered one week later, was identical in content and structure to that of the immediate posttest.

#### Dependent measures and data analyses

For each participant, the number of learning trials invested to achieve mastery for all categories was recorded. For the ATC condition, the total number of learning trials included both single-classification trials and comparison trials. Each comparison trial completed was counted as one trial invested; although these trials contained two images, participants supplied only one answer and the outcome of the trial could only advance progress toward the set mastery criteria for the category whose label was presented as the target of the trial. We also measured the time invested in learning for each participant.

In the ATC condition, the number of times each particular category combination (e.g., Alexander-Benjamin) was triggered for a comparison trial by each participant was recorded and ranked by frequency. Kendall’s coefficient of concordance (*W*) was used to measure the extent to which participants agreed on the most confusable category pairs. The *W* coefficient was then linearly transformed to reveal the average Spearman’s correlation between all possible pairs of raters^53^.

We measured classification accuracies at immediate and delayed posttests, and also examined separately performance on exemplars seen in learning (old) vs. unseen (novel) exemplars. Our primary dependent variable was *learning efficiency—*a *rate* obtained by dividing accuracy gains by learning investment. Efficiency measures are useful when mastery criteria are used, as they incorporate into a single measure both variations in trials (or time) to criterion and posttest performances (e.g.,^29^). In the present work, we calculated two types of efficiency scores based on two different measures of learning investment: trial-based efficiency and time-based efficiency. We multiplied our trial-based efficiency scores by 100 to obtain a learning rate, with the resulting number giving the percent gain in accuracy at either the immediate or delayed posttest per 100 trials invested in learning. Time-based efficiencies were multiplied by 10 to reflect the percent gain in accuracy for every ten minutes invested in learning. Condition differences were evaluated using standard parametric statistics with alpha set at 0.05 and 95% confidence intervals reported for each mean difference.

### Results

#### Learning investments

The ATC condition required significantly fewer learning trials to reach mastery (*M* = 345.95, *SD* = 76.41) than did the No Comparisons condition (*M* = 405.58, *SD* = 126.73). This difference of about 15% in trials to criterion was reliable,* t*(74) = 2.48, *p* = 0.015, *Cohen’s d* = 0.57, 95% Confidence Interval (CI) for the condition difference = [11.80, 107.46]. Time taken to reach the mastery criteria also significantly differed between condition with those in the ATC condition taking less time to reach mastery (*M* = 29.28 min., *SD* = 7.82) relative to the No Comparisons condition (*M* = 35.60 min., *SD* = 12.62), *t*(74) = 2.63, *p* = 0.010, *d* = 0.60**,** 95% CI = [1.53, 11.12].

#### Efficiency measures

##### Trial-based efficiency

**Figure 3:**
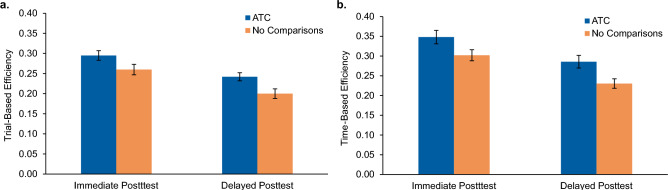
Efficiency results for Experiment 1. Immediate and delayed posttest efficiency scores are shown for the Adaptively Triggered Comparisons (ATC) condition and No Comparisons condition. Trial-based efficiency scores (**a**) indicate mean accuracy gain per 100 learning trials invested. Time-based efficiency scores (**b**) indicate mean accuracy gain per 10 min invested. Error bars represent ± 1 standard error of the mean.

Figure 3a shows the mean trial-based efficiency scores for both conditions at each posttest phase. At the immediate posttest, the ATC condition yielded a higher efficiency (*M* = 0.30, *SD* = 0.07) than the No Comparisons condition (*M* = 0.26, *SD* = 0.08), such that for every 100 learning trials invested, the average gain in immediate posttest accuracy was 30% for participants in the ATC condition and 26% in the No Comparisons condition. This difference bordered on significance,* t*(74) = − 1.99, *p* = 0.050, *d* = 0.53, 95% CI = [− 0.067, 0].

At the delayed posttest, efficiency reliably favored the ATC condition (*M* = 0.24, *SD* = 0.06) relative to the No Comparisons condition (*M* = 0.20, *SD* = 0.07), such that for every 100 learning trials invested, the average gain in delayed posttest accuracy was 24% in the ATC condition and 20% in the No Comparisons condition, *t*(74) = − 2.57, *p* = 0.012,* d* = 0.61, 95% CI = [− 0.072, − 0.009]. No interaction between condition and posttest phase was found, *F*(1, 74) = 0.53, *p* = 0.469.

##### Time-based efficiency

Figure 3b shows the mean time-based efficiencies for both conditions at each posttest phase. As with trial-based efficiency, the ATC condition yielded a higher time-based efficiency (*M* = 0.36, *SD* = 0.09) than the No Comparisons condition (*M* = 0.30, *SD* = 0.09) at the immediate posttest, such that the accuracy gain for every 10 min invested in learning was 36% for those in the ATC condition and 30% for those in the No Comparisons condition, *t*(74) = − 2.58, *p* = 0.012, *d* = 0.67, 95% CI = [− 0.09, − 0.01].

At the delayed posttest, efficiency once again favored the ATC condition (*M* = 0.29, *SD* = 0.09) relative to the No Comparisons condition (*M* = 0.23, *SD* = 0.08), such that for every ten minutes invested the average gain in delayed posttest accuracy was 29% in the ATC condition and 23% for participants in the No Comparisons condition, *t*(74) = − 3.27, *p* = 0.002, *d* = 0.70, 95% CI = [− 0.10, − 0.02]. There was no reliable interaction between condition and posttest phase, *F*(1, 74) = 0.50, *p* = 0.482.

#### Accuracy measures

**Figure 4:**
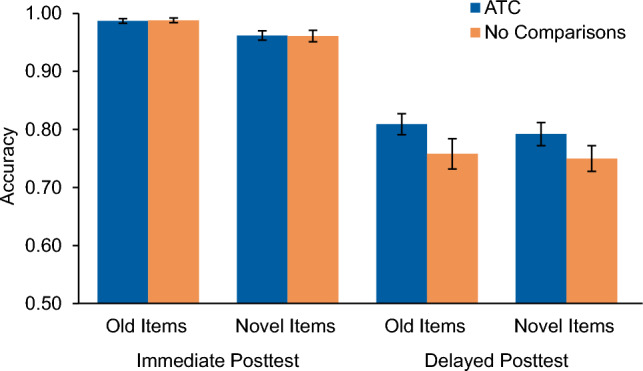
Accuracy results for Experiment 1. Immediate and delayed posttest accuracy scores are divided by old and novel items for the Adaptively Triggered Comparisons (ATC) condition and No Comparisons condition. Error bars represent ± 1 standard error of the mean.

At the posttests, all participants demonstrated an ability to classify the items previously seen in learning, as well as novel instances. Figure 4 depicts the average accuracy for each item type for both conditions. Looking at assessment accuracy across all items, immediate posttest accuracy did not differ between conditions (ATC: *M* = 0.97, SD = 0.03; No Comparisons:* M* = 0.97, *SD* = 0.04), t(74) = − 0.16, *p* = 0.875, *d* = 0, 95% CI = [− 0.02, 0.01]. Given that all participants learned to the same mastery criteria, we did not expect to see differences between conditions. Assessment accuracy was then divided into old and novel items and performance between conditions was compared. While old items were classified more accurately than novel items in both groups, there was no reliable evidence of a condition difference for classification accuracy on the old items (ATC: *M* = 0.99 *SD* = 0.03, No Comparisons: *M* = 0.99, *SD* = 0.02), *t*(74) = − 0.21, *p* = 0.839, *d* = 0.04, 95% CI = [− 0.01, 0.01], or on novel items (ATC: *M* = 0.96, *SD* = 0.05, No Comparisons: *M* = 0.96, *SD* = 0.06), *t*(74) = − 0.10, *p* = 0.922, *d* = 0.02, 95% CI = [− 0.03, 0.02].

At the delayed posttest, assessment accuracy was higher in the ATC condition (*M* = 0.80, *SD* = 0.11) than the No Comparisons condition (*M* = 0.75, *SD* = 0.14); however, this difference did not reach statistical significance,* t*(74) = − 1.61, *p* = 0.111, *d* = 0.39, 95% CI = [− 0.10, 0.01]. When the assessment was divided into old and novel items, classification accuracy favored the ATC condition for both item types, but neither difference reached significance (Old Items, ATC: *M* = 0.81, *SD* = 0.11, No Comparisons: *M* = 0.76, *SD* = 0.16, *t*(74) = − 1.59, *p* = 0.117, *d* = 0.37, 95% CI = [− 0.11, 0.01]; Novel Items, ATC: *M* = 0.79, *SD* = 0.12, No Comparisons: *M* = 0.75, *SD* = 0.13, *t*(74) = − 1.42, *d* = 0.33, 95% CI = [− 0.10, 0.02]).

#### Adaptive comparisons

The average number of comparison trials triggered by a participant in the ATC Condition was 31.71 (*SD* = 15.78) including an average of 18.05 (*SD* = 6.14) different category combinations. Following their initial confusion, participants recurrently triggered the same two categories for comparison an average of 0.70 (*SD* = 0.38) more times throughout the course of learning.

A chi-square test of independence determined that there was reliable non-zero agreement in confusability rankings among participants, *X*^2^ (230, *N* = 38) = 1293.93, *p* < 0.001. However, the coefficient of concordance indicated that the extent of this agreement was small, *W* = 0.15, and the resulting Spearman correlation coefficient revealed the relationship between participants’ rankings to be weak, *r*_*s*_ = 0.13.

## Experiment 2

Results from Experiment 1 showed that the inclusion of ATC trials resulted in improvements in efficiency both in terms of trials and time invested, with medium effect sizes. Provided with an opportunity to confront their confusions shortly after making them, participants were able to progress through the learning phase with fewer errors, reducing the amount of time and trials needed to achieve mastery, and without hindering performance in the short or long term.

From these results alone, it is not possible to determine whether the ATC benefit was due to the adaptive nature of the trials or simply to giving participants some opportunity to engage in simultaneous comparisons. To more directly test the role of adaptivity, Experiment 2 introduced a new *Non-Adaptive Comparisons (NAC)* condition in which participants engaged in roughly equal numbers of simultaneous comparison trials in learning as the ATC condition, but without regard to any specific confusions.

### Method

#### Participants

Given the effect sizes observed in Experiment 1, as well as modifications to the category retirement procedure (described below), a smaller sample of participants was recruited for Experiment 2. Sixty-two undergraduate psychology students completed the experiment; however, two participants were excluded for failing to follow experimenter instructions. The remaining participants were equally split between the ATC and NAC conditions. Participants provided informed consent and received partial course credit for their participation. This study was approved by the UCLA Institutional Review Board. All methods were carried out in accordance with their guidelines and regulations.

#### Design and procedure

Participants were randomly assigned to either the ATC condition or NAC condition. The ATC condition was identical to that used in Experiment 1. For the NAC condition, we aimed to keep the proportion of comparison trials to single-target learning trials consistent with the proportion that naturally arises in the ATC condition. Based on Experiment 1 and additional pilot data, we estimated that an ATC participant performing at the average in terms of learning trials invested would encounter approximately one comparison trial for every 8 single-item classification trials. Using this, the NAC condition was set to insert a comparison trial on every ninth learning trial.

The categories in the NAC condition were not based on learning confusions. Instead, whichever category was due to show up after the eighth single-classification trial (as determined by the ARTS sequencing) was then included in the comparison trial as the target, and the competing exemplar was chosen randomly from all other 21 categories.

All participants continued in the learning phase until all categories were mastered. However, rather than retaining all categories in the learning phase from start to finish, the learning phase was adjusted such that once a category was mastered, it was retired from the learning set. Retired categories only re-emerged when necessary as filler items to achieve correct spacing intervals for the remaining, unmastered categories. This decision was made to address concerns of overlearning for categories mastered early on, as large numbers of participants achieved ceiling performance on the immediate posttest (*n* = 33) and delayed posttest (*n* = 3) in Experiment 1. Following learning, participants completed an immediate and one-week delayed posttest.

### Results

#### Learning investments

The ATC condition required significantly fewer learning trials to reach the mastery criteria (*M* = 256.30, *SD* = 65.38) than the NAC condition (*M* = 329.17, *SD* = 90.81), *t*(58) = 3.57; *p* = 0.001, *d* = 0.92, 95% Confidence Interval (CI) for the condition difference = [31.97, 113.76]. Time taken to reach the mastery criteria was also reliably less for participants in the ATC condition (*M* = 26.67 min., *SD* = 9.54) than in the NAC condition (*M* = 34.27 min., *SD* = 11.81), *t*(58) = 2.74, *p* = 0.008, *d* = 0.71, 95% CI = [2.05, 13.15].

#### Efficiency measures

##### Trial-based efficiency

**Figure 5:**
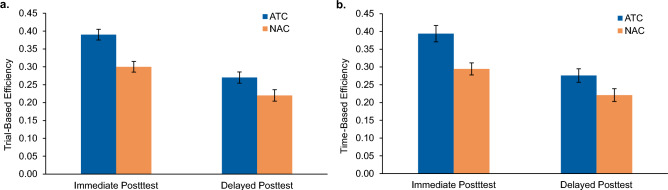
Efficiency results for Experiment 2. Immediate and delayed posttest efficiency scores are shown for the Adaptively Triggered Comparisons (ATC) condition and Non-Adaptive Comparisons (NAC) condition. Trial-based efficiency scores (**a**) indicate mean accuracy gain per 100 learning trials invested. Time-based efficiency scores (**b**) indicate mean accuracy gain per 10 min invested. Error bars represent ± 1 standard error of the mean.

Figure 5a shows the average trial-based efficiency by condition and posttest phase. The ATC condition yielded higher efficiency at both posttests. At the immediate posttest, participants in the ATC condition saw an average accuracy increase of 39% per 100 learning trials invested (*M* = 0.39, *SD* = 0.08), vs. a 30% increase (*M* = 0.30, *SD* = 0.08) in the NAC condition. This difference was highly reliable, *t*(58) = − 4.21; *p* < 0.001, with a large effect size, *d* = 1.13, 95% CI = [− 0.13, − 0.05].

At the delayed posttest, efficiency once again reliably favored the ATC condition with an average accuracy increase of 27% per 100 learning trials invested (*M* = 0.27, *SD* = 0.09) relative to the NAC condition which yielded an accuracy gain of 22% per 100 trials invested, (*M* = 0.22, *SD* = 0.09), *t*(58) = − 2.19; *p* = 0.033, and medium effect size, *d* = 0.56, 95% CI = [− 0.094, − 0.004].

##### Time-based efficiency

Figure 5b depicts the average time-based efficiency across conditions and posttest phases. At the immediate posttest, time-based efficiency for the ATC condition (*M* = 0.39, *SD* = 0.13) exceeded the NAC condition (*M* = 0.29, *SD* = 0.09), such that for every ten minutes invested in learning, immediate posttest accuracy improved 39% for the ATC condition and only 29% for the NAC condition. An independent samples t-test determined this difference to be reliable with a large effect size, *t*(58) = − 3.49, *p* < 0.001, *d* = 0.91, 95% CI = [− 0.16, − 0.04].

At the delayed posttest, time-based efficiency also reliably favored the ATC condition (*M* = 0.28, *SD* = 0.11) over the NAC condition (*M* = 0.22, *SD* = 0.10), such that for every ten minutes invested in learning, accuracy on the delayed posttest increased 28% for those in the ATC condition and only 22% for those in the NAC condition, *t*(58) = − 2.09, *p* = 0.041, *d* = 0.55, 95% CI = [− 0.108, − 0.002].

#### Accuracy measures

**Figure 6:**
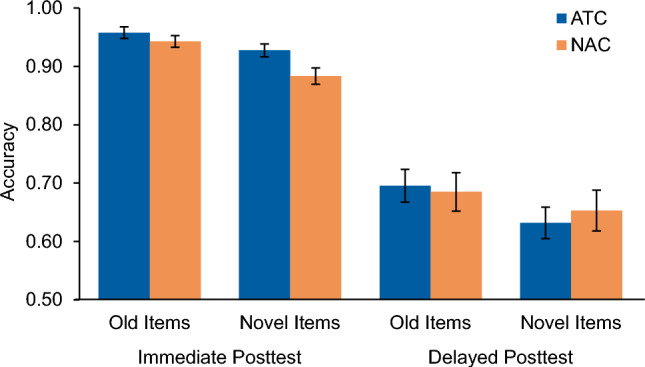
Accuracy results for Experiment 2. Immediate and delayed posttest accuracy scores are divided by old and novel items for the Adaptively Triggered Comparisons (ATC) condition and Non-Adaptive Comparisons (NAC) condition. Error bars represent ± 1 standard error of the mean.

All participants demonstrated an ability to classify both old and novel exemplars on the posttest assessments. Figure 6 depicts the average accuracy for each item type for both conditions. Immediate posttest accuracy across all items reliably varied by condition with those in the ATC condition scoring higher (*M* = 0.94, *SD* = 0.05) than those in the NAC condition (*M* = 0.91, *SD* = 0.06), *t*(58) = − 2.03; *p* = 0.047, *d* = 0.54, 95% CI = [− 0.0586, − 0.0005]. When divided into old and novel items, no reliable difference was found between conditions for performance classifying old items (ATC: *M* = 0.96, *SD* = 0.06, NAC: *M* = 0.94, *SD* = 0.05), *t*(58) = − 1.07, *p* = 0.287, *d* = 0.27, 95% CI = [− 0.02, 0.01]; however, accuracy on novel items was found to significantly differ between conditions, such that those in the ATC condition classified novel items more accurately (*M* = 0.93, *SD* = 0.06) than those in the NAC condition (*M* = 0.88, *SD* = 0.08), *t*(58) = − 2.41, *p* = 0.019, *d* = 0.63, 95% CI = [− 0.08. − 0.01].

At the delayed posttest, accuracy across all items did not differ significantly between conditions (ATC: *M* = 0.66, *SD* = 0.14; NAC: *M* = 0.67, *SD* = 0.18), *t*(58) = 0.13, *p* = 0.898, *d* = 0.06, 95% CI = [− 0.08, 0.09]. There was also no reliable difference between conditions when the assessment was divided into old items only (ATC: *M* = 0.70, *SD* = 0.15, NAC: *M* = 0.68, *SD* = 0.18), *t*(58) = 0.25, *p* = 0.805, *d* = 0.07, 95% CI = [− 0.10, 0.08], or novel items only (ATC: *M* = 0.63, *SD* = 0.15, NAC: *M* = 0.65, *SD* = 0.19), *t*(58) = 0.48, *p* = 0.633, *d* = 0.12, 95% CI = [− 0.07, 0.11].

#### Adaptive comparisons

On average, participants in the ATC condition received 26.57 (*SD* = 17.82) comparison trials throughout learning including 16.47 (*SD* = 7.15) different category combinations. This works out to one comparison trial per 9.65 trials invested, very similar to the proportion used in the NAC condition of one comparison trial for every 9.00 trials invested. Once a given category-combination was shown in a comparison trial, participants in the ATC condition confused the same two categories again an average of 0.54 times (*SD* = 0.37) throughout the remainder of the learning phase. Although the frequency and distribution of comparison trials varied by participant, overall, comparison trials occurred less often late in learning; 29.1% of all comparison trials were triggered within the first quarter of the learning phase, an additional 32.4% were triggered in the second quarter; 27.1% were triggered in the third; and only 11.4% were triggered in the final quarter.

A chi-square test of independence conducted on the ranked frequencies of category combinations demonstrated that agreement among participants was reliably different from zero, *X*^2^ (230, *N* = 30) = 1026.89, *p* < 0.001; however, the resulting coefficient of concordance and average Spearman’s correlation between participant rankings indicated that this agreement was weak, *W* = 0.15, *r*_*s*_ = 0.12.

## Discussion

We tested potential benefits of novel adaptively triggered comparisons in two experiments on facial identification, in the context of a basic adaptive learning system that guides category spacing in ways previously shown to outperform non-adaptive spacing^29,36^. Experiment 1 showed that adding comparison trials contingent on individual learner confusions improved learning relative to single-item classification, with medium effect sizes. Experiment 2 showed that this benefit was specific to the *adaptive* nature of the comparisons; learning was robustly enhanced (moderate to large effect sizes) by adaptively triggered comparisons relative to similar numbers of comparison trials whose content was not adapted to individual learners. Specifically, inclusion of ATC trials led to improved learning efficiency—mastery in shorter time—than conditions without comparison trials or with non-adaptive comparisons. This held true regardless of whether efficiency was calculated with respect to the total number of trials invested or the number of minutes invested in learning. Additionally, the results of Experiment 2 also suggested that ATCs may promote better transfer to novel instances of a category.

Notably, in the ATC conditions, the particular category pairs that triggered comparisons varied greatly across participants. Concordance tests showed very weak consistency across participants in individual confusions between pairs of facial identity categories. Across the two experiments, 212 of 231 possible (unordered) comparisons were triggered, and only two specific comparisons occurred for 50% or more of participants (one at 56% and another at 50%). Category similarity plays a meaningful role in determining the best ways to structure learning (e.g.,^25^). The weak concordance in triggered comparisons among learners indicates that “similarity” or confusability varies substantially across learners and underscores the value of an adaptive learning intervention that creates individualized opportunities for comparison.

Our data suggest that targeted comparison trials based on each individual learner’s performance were highly effective. Participants did not often recurrently trigger the same comparisons in the course of learning. Any given comparison was repeated on average 0.70 times for a participant in Experiment 1, and 0.54 times in Experiment 2. This outcome suggests remarkable effectiveness of a single comparison trial in allowing participants to discover and encode distinguishing information and avoid future confusions. A practical consequence of this result and the structure of the adaptive intervention is that trials involving particular category comparisons ceased once errors were no longer committed, preventing participants from engaging in ineffective comparisons and thus cutting down on the time and trials invested to achieve mastery.

The benefit of ATC trials may be particularly impressive considering that all learning conditions incorporated an effective, basic adaptive learning system. Given this backdrop, it is striking that adaptive comparisons provided learning advantages. Prior research suggests that on trials containing only a single exemplar, participants may still make comparisons between the currently presented item and the most recently viewed item^22,54^. The ARTS system used here implements spacing and interleaving^37^, such that missed items recur over shorter intervals, and exemplars from different categories are interleaved. As a result, commonly missed targets occur in close temporal proximity to many other items. These features alone may effectively promote between-category comparisons between unmastered categories without simultaneous comparison trials of any sort. The superiority of the ATC condition, then, indicates additional value from considering an individual learner’s data about missed targets and the incorrect answers given, and using this information to structure more effective comparison opportunities.

What is the likely nature of the benefits conferred by adaptively triggered comparisons? We believe these relate to complementary aspects of the *information* presented to the learner and also to the powerful influence of the *task* presented to learners and its effects in engaging underlying perceptual learning mechanisms. If, as many have theorized, perceptual learning mechanisms that lead to expertise in categorization involve the discovery of distinguishing features, these may be made more salient by simultaneous presentation. Perception of relations is facilitated by simultaneity, such that the information available may be effectively different from what is given by presentation of the same items at different times (cf.^18^). The foregoing may indicate the benefits of comparisons in general, as has been suggested in other research, (e.g.,^14^), but our results indicate that the benefits may be amplified by targeting categories that have already been shown through a given learner’s performance to be confusing.

Besides the availability of information, simultaneous comparison trials may engage underlying learning mechanisms, both perceptual and cognitive, in ways that differ from a succession of single item classification trials. This may be true for several reasons. One is that the format of a comparison trial may encourage search for and detection of information that differs between items (and hence their categories). As distinguishing features^1^ may be of greatest value in learning multi-category classifications, this trial format may promote search strategies that differ from information acquisition and selective encoding that develop from exposure to single instances. As with the information available in paired presentations, this task-provoked type of comparative processing may be most important where confusions are occurring. This idea is consistent with indications from some of our results that the ATC condition produced better transfer to novel instances (specifically, in the immediate posttest of Exp. 2). It may be useful to point out here that when we refer to “search strategies” that may be induced by one task format or another, we are not suggesting that these strategies, which may have implicit and explicit (intentional) components, are themselves the *results* of PL. Rather, certain task formats and related observer strategies may enhance PL, as in facilitating or impeding the discovery, selective weighting, and automatic pickup of distinguishing features (cf.^9^).

Another factor may be differences in cognitive load during learning. In single-item classification in our studies, learners had to consider a large number of possible response options. In contrast, comparison trials involved a single category label and two displays, perhaps allowing greater focus on a simpler task. In ongoing research, we have consistently found that comparison trials are completed more quickly than single-item classifications^55,56^ suggesting reduced load. Decreased cognitive load, relative to more standard classification trials, may free up resources for deeper processing of the visual information on a given comparison trial.

For all of these factors—the information made available, the engagement of learning processes by the task format, and the reduction of attentional or cognitive load—the present results indicate that tailoring comparisons to individual participant needs is particularly advantageous. Adaptivity in presenting comparisons may have enhanced learning both by personalizing which categories are to be compared and by presenting these comparisons at times in learning when they are needed most.

The present work tested comparisons in the domain of facial identity. Although the experimental questions are not unique to face perception, the use of faces as categories may involve some special considerations. Face categories are characterized by low within-category variability that may make transfer to novel instances relatively easy. Additionally, while the specific faces used in the present study were novel to all participants, adult humans may in general be considered experts in face perception and already know where to look for critical information. As a result of this learning history, facial identity categories may have reduced between-category similarity. If so, we might expect the benefits of ATCs to be even greater in other learning domains with higher between-category similarity. While we believe our observed results would generalize to other perceptual classification domains, the magnitudes of the effects could vary across categories and expertise of learners.

In sum, the studies reported here demonstrate that using learner performance to create adaptively triggered comparisons can enhance perceptual category learning. These results have important implications for our understanding of the learning benefits associated with comparisons, as well as for the development and design of learning technology that aims to optimize the acquisition of complex, visual categories.

## Data Availability

Data for both experiments reported here have been made available on OSF, https://osf.io/gt724/?view_only=5d5e3d33ab324a82aea3af17db7a577b.
